# A non-randomized comparison of gemcitabine-based chemoradiation with or without induction chemotherapy for locally advanced squamous cell carcinoma of the head and neck

**DOI:** 10.1186/1471-2407-9-273

**Published:** 2009-08-06

**Authors:** Pol M Specenier, Joost Weyler, Carl Van Laer, Danielle Van den Weyngaert, Jan Van den Brande, Manon T Huizing, Sevilay Altintas, Jan B Vermorken

**Affiliations:** 1 Antwerp University Hospital Antwerp, Department of Medical Oncology, Edegem, Belgium; 2 University of Antwerp, Epidemiology and Community Medicine, Edegem, Belgium; 3 Antwerp University Hospital, Department of Otolaryngology, Edegem, Belgium; 4 Antwerp University Radiotherapy, Antwerp, Belgium

## Abstract

**Background:**

Concomitant chemotherapy and radiotherapy (chemoradiation; CRT) is the standard treatment for locoregionally advanced squamous cell carcinoma of the head and neck (LA-SCCHN). CRT improves local control and overall survival (OS) when compared to radiotherapy (RT) alone. Induction chemotherapy (IC) reduces the risk of distant metastases (DM) and improves OS by 5% with the use of cisplatin/infusional 5 fluorouracil (PF) in meta-analysis. Adding a taxane to PF in the IC regimen confers a better outcome. Sequential treatment (ST) of IC followed by CRT is therefore under active investigation in multiple phase III trials.

**Methods:**

We compared the outcome of two cohorts of patients (pts) with LA-SCCHN treated at our institution with CRT (n = 27) or ST (n = 31), respectively. CRT consisted of GEM 100 mg/m^2 ^weekly + conventional RT (70 Gy); ST consisted of the same CRT preceded by platinum-based IC.

**Results:**

Response to IC: complete 8 (26%), partial 20 (65%), stable 1, progressive 1, not evaluable 1. Median follow up of the surviving pts: for CRT 73 months, for ST 51 months. Median time to distant metastasis (TDM) was for CRT 23.6 months, for ST not reached. Median OS was for CRT 20.2 months, for ST 40.2 months. Cox regression analysis, taking into account age, T and N stage and tumor site, showed a hazard ratio with ST of 1.190 for time to locoregional failure (p = 0.712), 0.162 for TDM (p = 0.002), and 0.441 for overall survival (OS) (p = 0.026).

**Conclusion:**

TDM and OS were found significantly longer in the ST cohort without a reduced locoregional control. Notwithstanding the limitations of a non-randomized single-center comparison, the results are in line with very preliminary data of randomized comparisons suggesting an improved outcome with ST.

## Background

Two thirds of the squamous cell carcinoma of the head and neck (SCCHN) are in a locoregionally advanced disease stage at time of diagnosis. Locoregionally advanced SCCHN is generally treated by a combination of chemotherapy and irradiation with or without surgery [1]. Concurrent chemotherapy and radiotherapy (chemoradiation) is widely adopted as standard of care for locoregionally advanced SCCHN after the publication of a large meta-analysis including individual data on 10.741 patients in 63 randomized trials [2,3]. Concurrent chemoradiation conferred an absolute survival benefit of 8% at 2 and 5 years. In contrast, the meta-analysis failed to demonstrate a survival advantage for induction chemotherapy followed by local treatment compared to local treatment alone. However when the meta-analysis was restricted to trials using the standard cisplatin/infusional 5-fluorouracil (PF) regimen, the absolute benefit at 5 years was 5% (p = 0.05). Recently, the addition of a taxane, docetaxel or paclitaxel, to cisplatin and 5-fluorouracil induction chemotherapy has shown to further improve response rates and survival outcomes when compared to the standard PF combination [4-6].

Moreover, induction chemotherapy reduces the risk of distant metastasis and offers the opportunity of assessing tumor chemosensitivity and selecting the patients who are candidate for organ preservation [7]. Sequential administration of induction chemotherapy followed by concurrent chemoradiation might combine the benefits of both [8]. Several cooperative groups are currently comparing this sequential approach to standard chemoradiation. However, definitive results of these randomized trials will not be available for several years. We therefore decided to perform a historical comparison of two cohorts of patients who were treated at our institution either by gemcitabine-based chemoradiation or induction chemotherapy followed by the same chemoradiation regimen.

## Methods

### Patients

Eligible patients were those with histologically confirmed locoregionally advanced SCCHN which were considered not to be amenable to surgery by a multidisciplinary dedicated team of head and neck surgeons, radiation oncologists and medical oncologists. Other criteria included age ≥ 18, World Health Organization performance status ≤ 2, adequate organ function, no prior chemotherapy and no radiotherapy above the clavicles, no evidence of other synchronous neoplasms, no evidence of distant metastases. Patients participated in in-house protocols from 1998 to 2006, of chemoradiation and/or induction chemotherapy regimens for which institutional review board approved informed consent was required.

### Treatment

All patients received weekly gemcitabine concurrently with radiation. Planned cumulative radiation dose was 70 Gy which was administered in 35 conventional fractions of 2 Gy over 7 weeks. Gemcitabine was started on the same day as the radiotherapy and was administered intravenously (dissolved in 150 ml NaCl 0.9%) over 30 minutes at a dose of 100 mg/m^2 ^within 2 hours before radiotherapy. Four patients in the sequential cohort received a lower gemcitabine dose (50 mg/m^2 ^in one patient and 10 mg/m^2 ^in three patients) while participating in an in-house protocol exploring serum levels of gemcitabine's metabolite difluorodeoxyuridine. In all cases gemcitabine was given for the duration of radiotherapy. In the sequentially treated cohort this same chemoradiation regimen was preceded by at least one cycle of cisplatin-based combination chemotherapy.

### Study design

This is a non-randomized comparison of two cohorts of patients treated at the Antwerp University Hospital by either a gemcitabine-based chemoradiation program or the same chemoradiation regimen preceded by cisplatin-based combination chemotherapy, the sequential treatment program. The objectives of this analysis were to compare the time to locoregional relapse, time to distant metastases, progression-free survival, overall survival and toxicities between these two cohorts. Time to locoregional failure (TLF) was defined as the time in months from the first day of treatment to the date locoregional relapse was recorded; time to distant metastases (TDM) was defined as the time in months from the first day of treatment to the date distant metastases were diagnosed; progression-free survival (PFS) was defined as the time in months from the first day of treatment to any form of progression event or death; overall survival time (OS) was defined as the time in months from the first day of treatment to the day of death.

### Assessments

Baseline tumor assessment was required within four weeks before registration as measured by computed tomography (CT) or magnetic resonance imaging (MRI) and direct endoscopy. Tumor response was assessed by repeating CT or MRI and endoscopy after two and four cycles of induction chemotherapy (or before chemoradiation in case less than four cycles were administered) and three months after the end of chemoradiation. World Health Organization criteria were used to determine response and disease progression. Endoscopy was performed at each visit at the multidisciplinary outpatient clinic. Imaging was repeated at any time disease progression was suspected.

### Statistical analysis

Crude comparison of time to event variables (TLF, TDM, PFS and OS) was based on Kaplan Meier analysis with log-rank testing. A multiple Cox regression analysis, taking into account age, T and N stage and tumor site, was used to calculate adjusted hazard ratios. All statistical tests comparing the two cohorts were two-sided using a significance level of α = 5%. The SPSS 13 software was used for all calculations.

## Results

Twenty-seven patients were treated in the concurrent chemoradiation cohort and 31 patients in the sequential cohort.

Patient and tumor characteristics are summarized in table 1 and 2. Median age was similar in both cohorts. There were slightly more male patients in the sequential cohort. Hypopharynx tumors predominated in the concurrent cohort wile oropharynx tumors constituted the largest group in the sequential cohort. Almost all tumors were stage IV in both cohorts while all patients had a good or excellent performance status.

**Table 1:** Patient and tumor characteristics

	CRT	ST	
Characteristics*	n = 27	n = 31	p
Age:			
median (range), years	57 (44-78)	58 (41-71)	0.208*
Gender			1
male	22	26	
female	5	5	
WHO performance status			0.7734
0	7	10	
1	20	21	
Primary tumor site:			0.179**
oropharynx	7	14	
hypopharynx	17	9	
larynx	1	4	
paranasal sinus	1	1	
nasopharynx		1	
unknown	1	2	
Stage:			**1
III	2	1	0.5931
IV	25	30	

CRT = concurrent chemotherapy and radiotherapy

ST = induction chemotherapy followed by CRT

*: Fischer's exact; **: Pearson chi-square; ^1^: T: p = 0.807; N: p = 0.163

**Table 2:** Primary tumor extent and nodal status

Concurrent treatment cohort					
	**N0**	**N1**	**N2**	**N3**	**All**
**Tx**				1	1
**T1**			1	1	2
**T2**			1	1	2
**T3**			4		4
**T4**	6	2	8	2	18

**All**	6	2	14	5	27

**Sequential treatment cohort**					

	**N0**	**N1**	**N2**	**N3**	**All**
**Tx**				2	2
**1**			1	1	2
**2**			1		1
**3**			6	1	7
**4**	4		13	2	19

**All**	4		21	6	31

### Concurrent chemoradiation cohort (CRT)

The patients in the CRT cohort received a median of 7 weekly cycles (range 2-8) of gemcitabine 100 mg/m^2 ^and a median cumulative radiation dose of 70 Gy. Radiation dose was 70 Gy in 22 of the 27 patients. Radiation was stopped at a cumulative dose of 66 Gy in 2 patients and at 68 Gy in 1 patient, either for toxicity or for practical reasons. Two patients received a higher dose (77.6 Gy and 84.75 Gy, respectively). Both patients had tumors which progressed while on treatment and went on with two daily fractions of 1.2 and 1.5 Gy, respectively. One patient refused further gemcitabine after 2 cycles of gemcitabine and continued with radiotherapy alone. Median treatment duration in the CRT cohort was 50 days (range: 37-56 days).

### Sequential treatment cohort (ST)

Patients in the ST received a median of 4 cycles of induction chemotherapy. Twenty-eight of the 31 patients received a taxane containing triplet which was TPF (cisplatin 75 mg/m^2 ^on day 1, docetaxel 75 mg/m^2 ^on day 1 and 5 fluorouracil 750 mg/m^2^/day as a continuous infusion on day 1 to 5 of a 3-weeks cycle) in 12 patients. In 3 of these patients TPF was associated with weekly cetuximab as part of a pilot study. Sixteen patients received DIP (docetaxel 60-75 mg/m^2 ^on day 1, cisplatin 50-75 mg/m^2 ^on day 1 or 5, and ifosfamide 1 g/m^2^/day as a continuous infusion on day 1 to 5 every 3 weeks). Two patients received the standard PF regimen and one patient was treated with carboplatin at an AUC of 5 and paclitaxel 175 mg/m^2^. Twenty-eight patients (90%) responded to induction chemotherapy. A clinical complete response was documented in eight patients (26%), a partial response in 20 (65%). The disease remained stable in one patient and progressed on induction chemotherapy in one. Response was not evaluable in one patient.

One patient in the ST cohort died after 2 administrations of gemcitabine and 18 Gy, due to grade 4 mucositis, grade 4 neutropenia and septic shock, which were treatment-related. In one patient, radiotherapy was stopped at 66 Gy because of toxicity and deteriorating general condition. One patient had progressive disease at the end of induction chemotherapy and received 80.5 Gy in 2 daily fractions of 1.2 Gy. The remaining 28 patients in the sequential cohort received the planned 70 Gy. Median duration of radiation treatment in the ST cohort was 49 days (range 10-53).

### Toxicities and survival

Toxicities are summarized in table 3. Both the hematologic and nonhematologic toxicities during chemoradiation scored higher in the ST cohort compared to the CRT cohort, apart from dermatitis which was unexpectedly more severe in the CRT cohort. A feeding tube was required during chemoradiation in 81% of the patients in the CRT cohort and in 97% of the patients in the ST cohort. Sixteen patients (59%) in the CRT cohort were alive without locoregional relapse after one year. None of these patients was feeding tube dependent at that point. Twenty-one (68%) were alive without locoregional relapse after 1 year in the ST cohort. Seven of them (33%) were still feeding tube dependent at that point.

**Table 3:** Toxicity

	CRT	ST
	
	n = 27	n = 31
	% (grade 3/4)	% (grade 3/4)
During induction chemotherapy		
Anemia	NA	100 (19)
Neutropenia	NA	94 (90)
Thrombocytopenia	NA	74 (13)
Mucositis	NA	13 (6)
Fatigue	NA	61 (3)
Diarrhea	NA	6 (3)
During chemoradiation		
Anemia	100 (4)	97 (16)
Neutropenia	41 (4)	58 (13*)
Thrombocytopenia	30 (0)	61 (6)
Mucositis	100 (81)	100 (97)
Dermatitis	100 (74)	100 (42)
Weigth loss > 5%	63	69
> 10%	30	28
Tube feeding during CRT	81	97
at 1 year**	0	33***

*: 1 patient grade 5; **in patients alive without locoregional recurrence

***: 4/10 oropharynx and 3/5 hypopharynx; NA = not applicable

**Figure 1:**
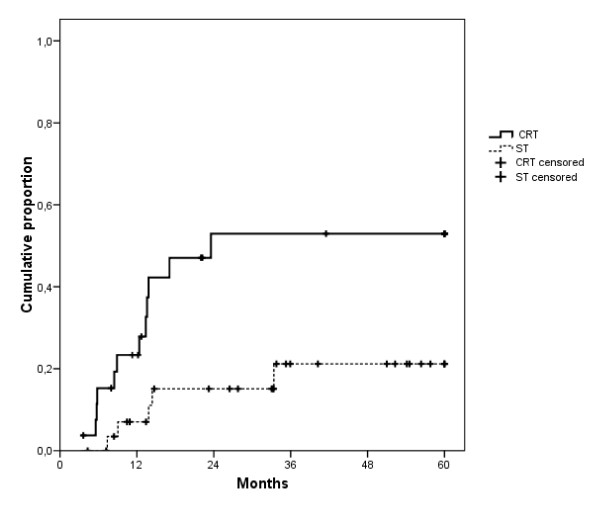
**Time to distant metastasis**. Median: CRT: 23.6 months; ST: not reached; Cox regression analysis p = 0.002

**Figure 2:**
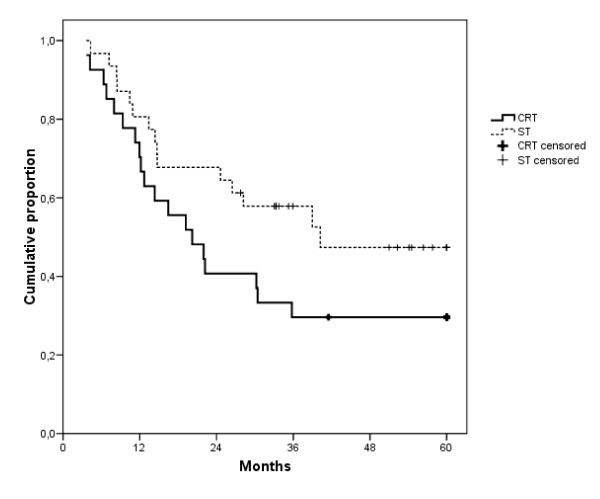
**Overall survival**. Median: CRT: 20.2 months; ST: 40.2 months; Cox regression analysis p = 0.026

Time to distant metastasis and overall survival are shown in figure 1 and 2, respectively. Median follow-up of the surviving patients was 73 months in the CRT cohort and 51 months in the ST cohort. We therefore censored all patients at 60 months of follow-up. The median TDM was 23.6 months with CRT and not reached with ST (log rank p = 0.010). Median OS was 20.2 months with CRT and 40.2 months with ST (log rank p = 0.100). However, multiple Cox regression analysis, taking into account age, T and N stage and tumor site, showed a hazard ratio with ST of 1.190 for TLF (p = 0.712), 0.162 for TDM (p = 0.002), and 0.441 for OS (p = 0.026).

## Discussion

Admittedly, the results of our analysis are to be interpreted with extreme caution as this is a non-randomized historical comparison of a relatively small number of patients treated at a single institution. Moreover, although the chemoradiation regimen was the same in both cohorts, it was not the currently widely accepted standard which is cisplatin-based. However, gemcitabine is a potent radiosensitizer which ensures excellent local control rates when added to radiation as treatment for locally advanced head and neck cancer, albeit at the cost of acute and late toxicity in excess of what is usually seen with cisplatin-based chemoradiation [9]. The median overall survival in our CRT cohort (20.2 months) was very similar to that observed in the chemoradiation arm of the Intergroup Study conducted in patients with unresectable squamous cell carcinoma of the head and neck (19.1 months) [10], at least suggesting that the difference between the two cohorts in our comparison was not due to a worse than expected outcome in the CRT cohort. In that Intergroup Study the standard cisplatin regimen (100 mg/m^2 ^on day 1, 22 and 42) was used. The locoregional control rate in our patients was promising and not significantly affected by the administration of induction chemotherapy. Induction chemotherapy did not preclude timely administration of radiotherapy, which is crucial as a protracted duration of radiation has a negative impact on treatment outcome [11-14]. The use of induction chemotherapy was associated with a statistically significant reduced risk of distant metastases. The reduction in distant metastasis translated into a better overall survival in a multiple Cox regression analysis taking into account age, T and N stage and tumor site. A reduced risk of distant metastasis has been a consistent observation in induction chemotherapy trials [2,15-17]. Graf et al [18] published the results of a non-randomised comparison of chemoradiation and sequential therapy performed at the Charité Medical School in Berlin. In contrast to our findings, local control was significantly worse in the sequential cohort. They observed a difference in overall survival in favor of the concomitant cohort which approached statistical significance. However, the radiation regimen in that study differed between the two cohorts: patients in the chemoradiation cohort received a hyperfractionated course up to 72 Gy while the patients in the sequential cohort received conventionally fractionated chemotherapy up to 70 Gy. Moreover, mean radiation dose achieved in the sequential cohort (65.3 Gy) was substantially lower than in the chemoradiation cohort (71.6 Gy). Finally, patients in the sequential cohort received only two cycles of PF induction chemotherapy and only one additional course of chemotherapy during radiation.

In our study, patients received a median of 4 cycles of chemotherapy which was a taxane containing triplet in 93%. Indeed, taxane containing triplets have demonstrated clear superiority over PF when administered as induction regimen. In our patients, induction chemotherapy did not jeopardize the subsequent radiation therapy as the median cumulative dose was 70 Gy in both cohorts. All these factors taken together may explain the discordant results of the analysis by Graf et al and ours.

Sequential treatment is associated with an increased toxicity. Hematological toxicity dominates during the induction phase of the therapy. Some of the hematological toxicity carries over to the chemoradiation phase. Of greater concern is the difference of long term feeding tube dependency rate between the two cohorts. Admittedly, the numbers in our study are small and the outcome might be different if cisplatin is used instead of gemcitabine. Nevertheless, we think this observation is noteworthy.

Thus far, the sequential administration of induction chemotherapy followed by chemoradiation should still be considered experimental and should not be used outside clinical trials. The short term feasibility of this paradigm has been demonstrated both in phase II and in phase III trials, but long term toxicity data are still lacking. Moreover, the superiority of this approach over chemoradiation alone in terms of efficacy has not been demonstrated in a phase III direct comparison. At least four large randomized phase III trials comparing TPF induction chemotherapy followed by chemoradiation to chemoradiation alone are currently planned or underway [7]. Data of one of these phase III trials were presented by Hitt et al [19]. TPF followed by irradiation and concurrent cisplatin 100 mg/m^2 ^every 3 weeks was associated with a higher CR rate and a better time to treatment failure than the same concurrent chemoradiation regimen alone. Paccagnella presented the final results of the phase II portion of an Italian randomized trial which compares TPF followed by irradiation in combination with cisplatin and 5-FU or the same chemoradiation alone [20]. TPF induction chemotherapy did not compromise the delivery of the subsequent chemoradiation and did not increase the incidence of severe radiation dermatitis or mucositis. The higher CR rate in the sequential arm justified the initiation of the phase III portion of the study.

## Conclusion

In conclusion, our non-randomized comparison mirrors the outcome of randomized trials which demonstrated a reduced risk of distant metastasis with induction chemotherapy in LA-SCCHN. In a multiple Cox regression analysis, this reduced risk of distant metastasis translated in a better overall survival. Locoregional control was not significantly affected by induction chemotherapy. Notwithstanding the limitations of a non-randomized single-centre comparison, the results are in line with the very preliminary data of randomized comparisons, mentioned above. Nevertheless, we suggest that the outcome of the large randomized trials need to be awaited before the sequential approach of induction chemotherapy followed by chemoradiation can replace cisplatin-based chemoradiation as the new standard treatment.

## Competing interests

JBV participated in Advisory Boards on Head and Neck Cancer of Merck-Serono, Amgen, Sanofi-Aventis, Lily, Boehringer-Ingelheim and Gentach and received honoraria from Merck-Serono and Sanofi-Aventis for scientific presentations.

The other authors have no other relevant affiliations or financial involvement with any organization or entity with a financial interest in or financial conflict with the matters discussed in the manuscript.

## Authors' contributions

PS conceived the study, collected and analyzed the data, participated in its design and coordination, and wrote the manuscript. JW performed the statistical analysis. CV, DVDW, JVDB, MTH, and SA contributed to the data acquisition and critically reviewed the manuscript. JBV participated in the design of the study, in its coordination and analysis and in writing the manuscript. All authors read and approved the final manuscript.

## Pre-publication history

The pre-publication history for this paper can be accessed here:

http://www.biomedcentral.com/1471-2407/9/273/prepub
